# Mucormycosis: An Unanticipated Progeny of COVID-19

**DOI:** 10.31729/jnma.6686

**Published:** 2021-09-30

**Authors:** Gentle Sunder Shrestha, Sabin Bhandari, Ritesh Lamsal, Urmila Gurung

**Affiliations:** 1 Department of Anaesthesiology, Tribhuvan University Teaching Hospital, Maharajgunj, Kathmandu, Nepal; 2 Department of Otolaryngology-Head and Neck Surgery, Tribhuvan University Teaching Hospital, Maharajgunj, Kathmandu, Nepal

**Keywords:** *COVID-19*, *mucormycosis*, *Nepal*

## Abstract

The rapid surge of COVID-19 cases in the second wave of the pandemic has crippled the healthcare delivery system in Nepal and neighboring countries. Unlike in the first wave of the pandemic, several cases of mucormycosis have been reported in patients with COVID-19 from Nepal and India. In this report, we briefly describe the clinical presentation, diagnosis, and risk factors for mucormycosis and explore why patients with COVID-19 are at an increased risk for developing the infection. As treatment of mucormycosis is challenging and consumes a lot of resources, prevention of mucormycosis is pivotal in low-income countries like Nepal. We also highlight some basic steps that are easy to perform and important to reduce the risk of infection.

## INTRODUCTION

Coronavirus disease 2019 (COVID-19) pandemic caused by severe acute respiratory syndrome coronavirus 2 (SARS-CoV-2) is associated with a wide range of disease patterns, from mild to life-threatening pneumonia to opportunistic bacterial and fungal co-infections.^1^ Though aspergillosis and candidiasis are the most commonly reported fungal infections in patients with COVID-19,^2^ mucormycosis is being reported at an unprecedented level.

A complex interplay of factors including poor glycemic control, acidic medium (metabolic acidosis, diabetic ketoacidosis), high iron level and decreased phagocytic activity of white blood cells (WBC) due to immunosuppression along with prolonged hospitalization with or without mechanical ventilation might facilitate mucormycosis.^3^

## CLINICAL PRESENTATION AND DIAGNOSIS

Mucormycosis is an angioinvasive disease caused by fungi of the order Mucorales. The genera commonly implicated in human infections include Rhizopus, Mucor, Rhizomucor, Cunninghamella, and Absidia.^4^ The most common among these is the Rhizopus species, which is responsible for nearly 46% of mucormycosis cases in humans.^5^ Rhino-orbital-cerebral mucormycosis and pulmonary mucormycosis are life-threatening infections caused by these fungi. Gastrointestinal, pulmonary, renal, isolated central nervous system, cutaneous, and disseminated mucormycosis are less common clinical presentations of mucormycosis.

The diagnosis is established by a high index of suspicion from the clinical presentation, recognition of host factors, radiologic findings, and microscopic identification of the organism with the typical structure of a Mucorales.^6^ Symptoms of rhino-orbital mucormycosis include any combinations of unilateral retro-orbital pain, orbital swelling, ptosis, diplopia, decreased vision, facial pain, decreased sensation over cheek, nasal blockage, nasal discharge, blackish discoloration on the face or hard palate. Neurological symptoms will be seen if it has spread intracranially. Tissue necrosis is the hallmark of mucormycosis and is seen as black eschar but may not be found in the early stages. There are no specific biologic markers to identify mucormycosis. Culture report in mucormycosis is usually negative.^6^ Known risk factors for mucormycosis before the COVID-19 pandemic were diabetes mellitus, hematological malignancy, organ transplantation, autoimmune disorders, immunosuppressant use, iron overload, burns, trauma, and prior treatment with antifungals mainly voriconazole.^7^

## RISK FACTORS AND POSSIBLE PATHOGENESIS

The reason why mucormycosis is common in patients with COVID-19 remains largely speculative. An analysis of 101 cases of mucormycosis in patients with COVID-19 found hyperglycemia at presentation due to pre-existing or new-onset diabetes mellitus, new-onset hyperglycemia, or diabetic ketoacidosis to be the single-most important risk factor in the majority of cases (83.3%).^3^ Nearly 76% of the patients had taken corticosteroids for COVID-19, followed by remdesivir (20%) and tocilizumab (4%).

It is hypothesized that immune dysregulation associated with COVID-19, such as reduced numbers of T lymphocytes, CD4+T, and CD8+T cells, may alter innate immunity,^8^ thus favoring fungal growth. Similarly, the pro-coagulable state associated with COVID-19^9^ causes thrombosis of feeding arteries leading to necrosis of tissues and secondary invasion by the fungus.

The rampant use of high-dose steroids without proper monitoringofbloodglucoselevelsresultsinuncontrolled hyperglycemia andcan precipitate diabetes ketoacidosis. The resultant acidosis acts as a fertile medium for mucor spores to germinate. The concurrent use of steroids and immunomodulatory drugs such as tocilizumab also reduces the phagocytic activity of white blood cells, making a patient with diabetes mellitus vulnerable to mucormycosis.^3,10^ Mucormycosis thrives on freely available iron. Hyperglycemia can cause glycosylation of transferrin and ferritin, reducing their iron affinity and increasing the free iron. Furthermore, the low pH caused by the accumulation of ketone bodies in diabetic ketoacidosis strongly impairs the ability of transferrin to chelate iron, thereby increasing free iron. Also, there is an increase in ferritin levels in patients with COVID-19 due to the surge of cytokines, especially interleukin-6, thus increasing free iron.^3,11^

Some authors have implicated other possible risk factors, like the use of monoclonal antibodies and the rampant use of broad-spectrum antibiotics as the cause for increase in new-onset fungal infections.^1^ Few cases reported in grey literature have speculated other possible reasons for the surge of mucormycosis, such as the supply of contaminated industrial oxygen to the hospitals, use of unsterile water in humidifier and steam inhaler, and intake of zinc. More data is needed to establish an association of these potential risk factors with mucormycosis.

## TREATMENT

The treatment of mucormycosis usually requires early extensive surgical debridement in combination with antifungal medications and rapid control of underlying medical condition. Generally, a multidisciplinary team consisting of otorhinolaryngologists, neurosurgeons, ophthalmologists, anesthesiologists, intensivists, endocrinologists, infectious disease experts, microbiologists, and pathologists are involved in the treatment. Once mucormycosis is suspected clinically, antifungals may be initiated before the formal microbiolgical reporting. It is important to perform appropriate imaging and document the extent of the fungal invasion urgently to help prognosticate and also to guide surgical debridement. Liposomal amphotericin B is recommended as the first-line treatment. Intravenous isavuconazole and intravenous or delayed-release oral posaconazole are recommended with moderate strength as first line treatment if patient is intolerant or refractory to amphotericin B and strongly recommended as salvage therapy. Use of amphotericin B deoxycholate is discouraged whenever alternative therapies are available, because of substantial nephrotoxicity, specifically in the doses and treatment durations needed for mucormycosis. The optimal duration of therapy in mucormycosis is not clear and is guided by the clinical response, tolerability, and radiographic improvement. Even with the best treatment, all-cause mortality ranges from 40% to 80% depending on underlying conditions and sites of infection.^12^ Cerebral involvement is a significant predictor of mortality in patients with mucormycosis.^13^

As the cost of liposomal amphotericin is high and long-term therapy is needed for cure, treatment of mucormycosis is difficult in Nepal. There are added constraints, such as an acute shortage of the drug. Furthermore, the management of many cases with invasive disease requires a high level of expertise, and a multidisciplinary collaboration, which is not readily available in most parts of the country. Many patients are referred to hospitals in large cities as surgical treatment of patients with COVID-19 is not readily available in Nepal. This not only delays the initiation of therapy but also carries the risk of spread of COVID-19 patients need to travel long distances, often without adequate infection prevention measures. It is not uncommon to have mucormycosis during active stage of COVID-19.

## WAY FORWARD

During the second wave of the COVID-19 pandemic in Nepal, several cases of mucormycosis have been reported throughout the country. Invasive mucormycosis is difficult to treat and requires exhaustive resources. In resource-limited settings like ours, it is extremely important to adopt all possible measures to prevent mucormycosis in patients with COVID-19. There should be a high index of suspicion of fungal infection in patients with COVID-19 so that timely diagnosis and early initiation of treatment can be done. Evidence-based use of corticosteroids should be advocated and all efforts should be made to maintain normal glucose levels in these patients. Most patients with COVID-19 do not require high doses or prolonged treatment with corticosteroids.

Without clear evidence of benefit, drugs targeting immune pathways, such as tocilizumab, should be used with caution.^10^ The use of prophylactic broad-spectrum antibiotics and antifungals, without definite evidence of infection should be discouraged. All other therapeutic agents used should be monitored to achieve a therapeutic effect at the lowest dose and shortest duration possible. Often less-is-more.
